# Methanol{2-meth­oxy-6-[(2-oxidoprop­yl)imino­meth­yl]phenolato}dioxidomolyb­denum(VI)

**DOI:** 10.1107/S1600536809047485

**Published:** 2009-11-18

**Authors:** Samira Saeednia, Iran Sheikhshoaie, Helen Stoeckli-Evans

**Affiliations:** a Chemistry Department, Shahid Bahonar University, Kerman, Iran; b Institute of Physics, University of Neuchαtel, Rue Emile-Argand 11, CH-2009 Neuchâtel, Switzerland

## Abstract

In the structure of the title compound, [Mo(C_11_H_13_NO_3_)O_2_(CH_3_OH)], the Mo^VI^ ion is octahedrally coordinated by two oxide O atoms, the N atom and two deprotonated OH groups of the tridentate Schiff base ligand 2-meth­oxy-6-[(2-oxidoprop­yl)imino­meth­yl]phenolate and by a methanol O atom. In the crystal structure, two complexes are linked *via* O—H⋯O hydrogen bonds, yielding a centrosymmetric arrangement involving the methanol hydr­oxy group and one of the ligand O atoms coordinated to the Mo^VI^ ion.

## Related literature

For molybdenum (VI) Schiff base complexes in bioinorganic chemistry, see: Holm *et al.* (1996 ▶) and as oxidation catalysts, see: Arnaiz *et al.* (2000 ▶); Sheikhshoaie *et al.* (2009 ▶). For similar structures, see: Abbasi *et al.* (2008 ▶); Monadi *et al.* (2009 ▶); Syamal & Maurya (1989 ▶).
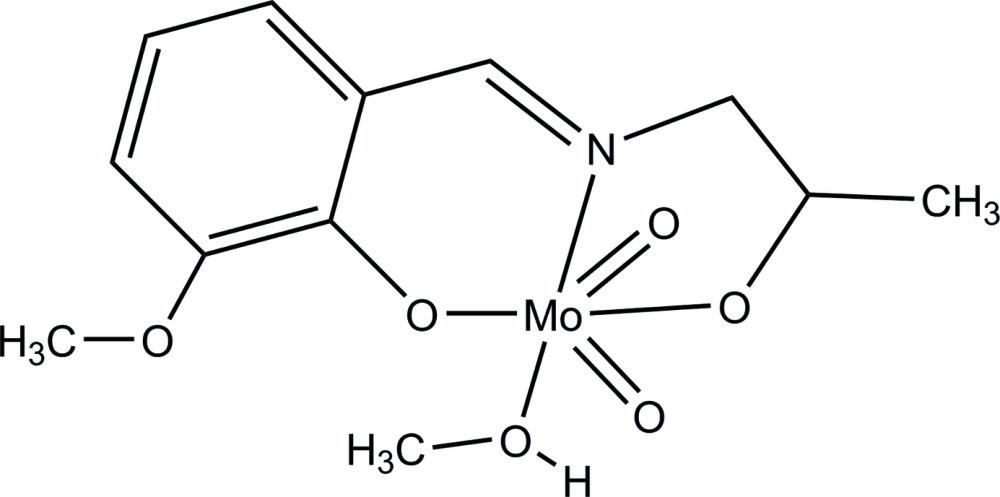


## Experimental

### 

#### Crystal data

[Mo(C_11_H_13_NO_3_)O_2_(CH_4_O)]
*M* *_r_* = 367.21
Monoclinic, 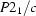

*a* = 6.7551 (5) Å
*b* = 15.8357 (14) Å
*c* = 13.1198 (10) Å
β = 98.287 (9)°
*V* = 1388.79 (19) Å^3^
*Z* = 4
Mo *K*α radiation
μ = 0.97 mm^−1^
*T* = 173 K
0.38 × 0.38 × 0.34 mm

#### Data collection

Stoe IPDS diffractometer
Absorption correction: multi-scan (*MULscanABS* in *PLATON*; Spek, 2009 ▶) *T*_min_ = 0.625, *T*_max_ = 0.716
10555 measured reflections
2666 independent reflections
2601 reflections with *I* > 2σ(*I*)
*R*_int_ = 0.035

#### Refinement

*R*[*F* ^2^ > 2σ(*F* ^2^)] = 0.024
*wR*(*F* ^2^) = 0.063
*S* = 1.16
2666 reflections
187 parameters
1 restraint
H atoms treated by a mixture of independent and constrained refinement
Δρ_max_ = 0.41 e Å^−3^
Δρ_min_ = −0.60 e Å^−3^

### 

Data collection: *EXPOSE* in *IPDS-I* (Stoe & Cie, 2000 ▶); cell refinement: *CELL* in *IPDS-I*; data reduction: *INTEGRATE* in *IPDS-I*; program(s) used to solve structure: *SHELXS97* (Sheldrick, 2008 ▶); program(s) used to refine structure: *SHELXL97* (Sheldrick, 2008 ▶); molecular graphics: *PLATON* (Spek, 2009 ▶) and *Mercury* (Macrae *et al.*, 2006 ▶); software used to prepare material for publication: *SHELXL97*.

## Supplementary Material

Crystal structure: contains datablocks I, global. DOI: 10.1107/S1600536809047485/fi2093sup1.cif

Structure factors: contains datablocks I. DOI: 10.1107/S1600536809047485/fi2093Isup2.hkl

Additional supplementary materials:  crystallographic information; 3D view; checkCIF report

## Figures and Tables

**Table 1:** Hydrogen-bond geometry (Å, °)

*D*—H⋯*A*	*D*—H	H⋯*A*	*D*⋯*A*	*D*—H⋯*A*
O6—H6*O*⋯O1^i^	0.83 (3)	1.81 (3)	2.639 (2)	176 (2)

Symmetry code: (i) 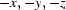.

**Fig. 1.**
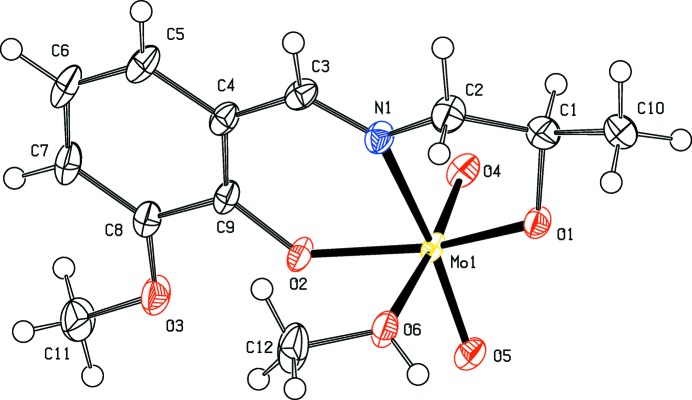
The molecular structure structure of the title compound, showing the numbering scheme and the thermal ellipsoids drawn at the 50% probability level.

**Fig. 2.**
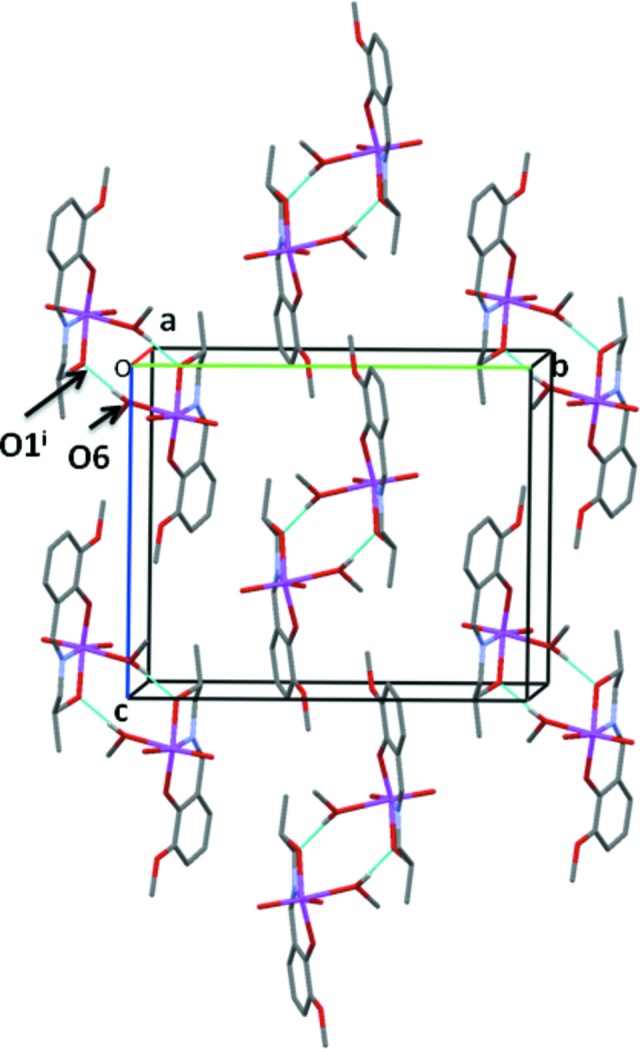
A view along the a axis of the crystal packing of the title compound, showing the formation of the O-H···O hydrogen bonded dimers [hydrogen bonds are shown as pale blue lines; H-atoms not involved in hydrogen bonding have been removed for clarity; symmetry operation (i) = -x, -y, -z].

## supplementary crystallographic information

### Comment

Various molybdenum(VI) Schiff base complexes have been studied due to their importance in the domains of bioinorganic chemistry (Holm *et al.*, (1996), analytical chemistry, oxidation catalyst (Arnaiz *et al.*, 2000; Sheikhshoaie *et al.*, 2009) and structural chemistry (Abbasi *et al.*, 2008; Monadi *et al.*, 2009; Syamal & Maurya, 1989). In continuation of our interest in this line of research we have prepared the title compound, synthesized by the reaction of MoO_2_(acac)_2_ and the Schiff base ligand 2-[(2-hydroxy-propylimino)-methyl]-phenol in methanol.

The molecular structure of the title compound is illustrated in Fig. 1 and geometrical parameters are available in the supplementary material as well as in the deposited CIF. The molybdenum atom, Mo1, has a distorted octahedral coordination, being coordinated by the N and two O-atoms of the tridentate Schiff base ligand (N1, O1 and O2), two oxido O-atoms (O4 and O5), and by the O-atom (O6) of the coordinating methanol molecule. The Mo—O and Mo—N bond distances are similar to those reported for the molybdenum (VI) Schiff base complex, {1,1'-[(2,2-Dimethylpropane-1,3-diyl)bis(nitrilomethylidyne)] di-2-naphtholato}dioxidomolybdenum(VI) dichloromethane 1.75-solvate, (Monadi *et al.*, 2009).

In the crystal, complexes are linked *via* hydrogen bonds, O6—H6O···O1^i^ [symmetry operation (i) = -x, -y, -z], involving the methanol hydroxy group and a ligand O-atom coordinating to the second Mo atom so forming centrosymmetric dimers (Table 1 and Fig. 2).

### Experimental

The title compound was prepared by adding MoO_2_(acac)_2_ (0.327 g) to a dry methanolic solution (30 ml) of 2-[(2-hydroxy-propylimino)-methyl]-phenol (0.209 g); a 1:1 equimolar ratio. The mixture was then refluxed for 5 h. On cooling a yellow crystalline powder formed, which were filtered off. Crystals of the title complex, suitable for X-ray analysis, were obtained as yellow blocks by slow evaporation at room temperature of a solution in methanol.

### Refinement

The OH H-atom was located in a difference electron-density map and refined with a distance restraint of 0.84 (2) Å and *U*_iso_(H) = 1.5*U*_eq_(parent O-atom). The remaining H atoms could all be located from difference electron-density maps but were included in calculated positions and treated as riding atoms: C—H = 0.95 - 1.00 Å, with *U*_iso_(H) = k × *U*_eq_(parent C-atom), where k = 1.2 for CH and CH_2_ H-atoms, and 1.5 for CH_3_ H-atoms.

### Crystal data

**Table:**

[Mo(C_11_H_13_NO_3_)O_2_(CH_4_O)]	*F*(000) = 744
*M**_r_* = 367.21	*D*_x_ = 1.756 Mg m^−^^3^
Monoclinic, *P*2_1_/*c*	Mo *K*α radiation, λ = 0.71073 Å
Hall symbol: -P 2ybc	Cell parameters from 8000 reflections
*a* = 6.7551 (5) Å	θ = 2.0–26.1°
*b* = 15.8357 (14) Å	µ = 0.97 mm^−^^1^
*c* = 13.1198 (10) Å	*T* = 173 K
β = 98.287 (9)°	Block, yellow
*V* = 1388.79 (19) Å^3^	0.38 × 0.38 × 0.34 mm
*Z* = 4	

### Data collection

**Table:**

Stoe IPDS diffractometer	2666 independent reflections
Radiation source: fine-focus sealed tube	2601 reflections with *I* > 2σ(*I*)
graphite	*R*_int_ = 0.035
φ rotation scans	θ_max_ = 25.9°, θ_min_ = 2.6°
Absorption correction: multi-scan (MULscanABS in *PLATON*; Spek, 2009)	*h* = −7→8
*T*_min_ = 0.625, *T*_max_ = 0.716	*k* = −19→19
10555 measured reflections	*l* = −16→16

### Refinement

**Table:**

Refinement on *F*^2^	Primary atom site location: structure-invariant direct methods
Least-squares matrix: full	Secondary atom site location: difference Fourier map
*R*[*F*^2^ > 2σ(*F*^2^)] = 0.024	Hydrogen site location: inferred from neighbouring sites
*wR*(*F*^2^) = 0.063	H atoms treated by a mixture of independent and constrained refinement
*S* = 1.16	*w* = 1/[σ^2^(*F*_o_^2^) + (0.0288*P*)^2^ + 1.2212*P*] where *P* = (*F*_o_^2^ + 2*F*_c_^2^)/3
2666 reflections	(Δ/σ)_max_ < 0.001
187 parameters	Δρ_max_ = 0.41 e Å^−^^3^
1 restraint	Δρ_min_ = −0.59 e Å^−^^3^

### Special details

**Table:**

Geometry. Bond distances, angles *etc*. have been calculated using the rounded fractional coordinates. All su's are estimated from the variances of the (full) variance-covariance matrix. The cell e.s.d.'s are taken into account in the estimation of distances, angles and torsion angles
Refinement. The OH H-atom was located in a difference electron-density map and refined with a distance constraint of 0.84 (2) Å and *U*_iso_(H) = 1.5*U*_eq_(parent O-atom). The remainder of the H-atoms could all be located from difference electron-density maps but were included in calculated positions and treated as riding atoms: C—H = 0.95 - 1.00 Å, with *U*_iso_(H) = k × *U*_eq_(parent C-atom), where k = 1.2 for CH and CH_2_ H-atoms, and 1.5 for methyl H-atoms. Using the Stoe IPDS1, one-circle image plate diffraction system, it is often only possible to access 94% maximum of the Ewald sphere depending on the crystal system and the position of the crystal. Here however, 98% of the data were accessible out to 25° in θ.

### Fractional atomic coordinates and isotropic or equivalent isotropic displacement parameters (Å^2^)

**Table:**

	*x*	*y*	*z*	*U*_iso_*/*U*_eq_	
Mo1	0.03631 (2)	0.11249 (1)	0.15344 (1)	0.0156 (1)	
O1	0.0326 (2)	0.12028 (9)	0.00507 (12)	0.0197 (4)	
O2	0.1700 (2)	0.08957 (10)	0.29193 (11)	0.0210 (4)	
O3	0.1839 (2)	0.06916 (11)	0.48988 (12)	0.0289 (5)	
O4	−0.0341 (2)	0.21363 (10)	0.17290 (12)	0.0236 (4)	
O5	−0.1751 (2)	0.05346 (10)	0.15022 (12)	0.0228 (4)	
O6	0.1883 (2)	−0.01628 (10)	0.12101 (12)	0.0238 (5)	
N1	0.3526 (3)	0.14577 (12)	0.13382 (14)	0.0202 (5)	
C1	0.1774 (3)	0.17293 (15)	−0.03365 (18)	0.0261 (6)	
C2	0.3783 (3)	0.14935 (16)	0.02534 (17)	0.0270 (7)	
C3	0.5000 (3)	0.16301 (13)	0.20319 (17)	0.0203 (6)	
C4	0.4983 (3)	0.15205 (13)	0.31189 (17)	0.0200 (6)	
C5	0.6718 (3)	0.17449 (15)	0.37898 (19)	0.0263 (7)	
C6	0.6822 (3)	0.16196 (17)	0.48262 (19)	0.0319 (7)	
C7	0.5213 (4)	0.12683 (16)	0.52236 (19)	0.0294 (7)	
C8	0.3482 (3)	0.10346 (14)	0.45824 (18)	0.0223 (6)	
C9	0.3363 (3)	0.11557 (12)	0.35135 (17)	0.0189 (6)	
C10	0.1682 (4)	0.16163 (15)	−0.14788 (18)	0.0272 (7)	
C11	0.1874 (4)	0.05673 (19)	0.59784 (18)	0.0359 (8)	
C12	0.3293 (4)	−0.06281 (18)	0.1874 (2)	0.0387 (8)	
H1	0.14830	0.23330	−0.01900	0.0310*	
H2A	0.42220	0.09380	0.00210	0.0320*	
H2B	0.48010	0.19220	0.01460	0.0320*	
H3	0.61850	0.18450	0.18150	0.0240*	
H5	0.78240	0.19850	0.35210	0.0320*	
H6	0.79980	0.17730	0.52760	0.0380*	
H6O	0.117 (4)	−0.0505 (16)	0.084 (2)	0.0360*	
H7	0.52970	0.11860	0.59460	0.0350*	
H10A	0.03800	0.18110	−0.18270	0.0410*	
H10B	0.18550	0.10180	−0.16340	0.0410*	
H10C	0.27490	0.19470	−0.17210	0.0410*	
H11A	0.20460	0.11130	0.63330	0.0540*	
H11B	0.29890	0.01940	0.62400	0.0540*	
H11C	0.06120	0.03100	0.61040	0.0540*	
H12A	0.27690	−0.07390	0.25200	0.0580*	
H12B	0.45390	−0.03050	0.20190	0.0580*	
H12C	0.35560	−0.11650	0.15480	0.0580*	

### Atomic displacement parameters (Å^2^)

**Table:**

	*U*^11^	*U*^22^	*U*^33^	*U*^12^	*U*^13^	*U*^23^
Mo1	0.0105 (1)	0.0213 (1)	0.0142 (1)	−0.0008 (1)	−0.0009 (1)	−0.0025 (1)
O1	0.0183 (7)	0.0248 (8)	0.0153 (7)	−0.0021 (6)	−0.0003 (6)	−0.0011 (6)
O2	0.0145 (7)	0.0326 (8)	0.0149 (7)	−0.0038 (6)	−0.0017 (6)	−0.0012 (6)
O3	0.0265 (8)	0.0427 (10)	0.0162 (8)	−0.0051 (7)	−0.0015 (6)	0.0021 (7)
O4	0.0205 (7)	0.0258 (8)	0.0236 (8)	−0.0017 (6)	0.0005 (6)	−0.0059 (6)
O5	0.0163 (7)	0.0257 (8)	0.0254 (8)	−0.0022 (6)	−0.0007 (6)	−0.0022 (6)
O6	0.0233 (8)	0.0236 (8)	0.0215 (8)	0.0028 (6)	−0.0067 (6)	−0.0039 (6)
N1	0.0142 (8)	0.0271 (10)	0.0193 (9)	−0.0012 (7)	0.0022 (7)	0.0005 (7)
C1	0.0287 (11)	0.0255 (11)	0.0239 (11)	−0.0032 (9)	0.0031 (9)	0.0022 (9)
C2	0.0212 (10)	0.0398 (13)	0.0206 (11)	−0.0048 (10)	0.0056 (9)	0.0007 (10)
C3	0.0120 (9)	0.0218 (10)	0.0271 (11)	0.0007 (8)	0.0027 (8)	−0.0034 (9)
C4	0.0142 (9)	0.0210 (10)	0.0237 (11)	0.0027 (8)	−0.0011 (8)	−0.0055 (8)
C5	0.0146 (10)	0.0322 (12)	0.0305 (12)	−0.0005 (9)	−0.0025 (9)	−0.0084 (10)
C6	0.0202 (10)	0.0413 (14)	0.0300 (13)	−0.0010 (10)	−0.0103 (9)	−0.0085 (11)
C7	0.0282 (12)	0.0373 (13)	0.0193 (11)	0.0023 (10)	−0.0076 (10)	−0.0032 (10)
C8	0.0212 (11)	0.0253 (11)	0.0190 (11)	0.0021 (8)	−0.0020 (9)	−0.0024 (8)
C9	0.0152 (10)	0.0201 (10)	0.0196 (11)	0.0035 (7)	−0.0039 (8)	−0.0051 (8)
C10	0.0303 (12)	0.0283 (11)	0.0228 (12)	−0.0026 (9)	0.0036 (9)	0.0054 (9)
C11	0.0410 (14)	0.0494 (16)	0.0166 (11)	−0.0071 (12)	0.0017 (10)	0.0023 (11)
C12	0.0356 (13)	0.0363 (14)	0.0382 (15)	0.0152 (11)	−0.0147 (11)	−0.0059 (11)

### Geometric parameters (Å, °)

**Table:**

Mo1—O1	1.9471 (16)	C6—C7	1.388 (3)
Mo1—O2	1.9431 (14)	C7—C8	1.389 (3)
Mo1—O4	1.7005 (16)	C8—C9	1.406 (3)
Mo1—O5	1.7022 (15)	C1—H1	1.0000
Mo1—O6	2.3493 (16)	C2—H2A	0.9900
Mo1—N1	2.251 (2)	C2—H2B	0.9900
O1—C1	1.433 (3)	C3—H3	0.9500
O2—C9	1.337 (3)	C5—H5	0.9500
O3—C8	1.354 (3)	C6—H6	0.9500
O3—C11	1.427 (3)	C7—H7	0.9500
O6—C12	1.403 (3)	C10—H10A	0.9800
O6—H6O	0.83 (3)	C10—H10B	0.9800
N1—C2	1.460 (3)	C10—H10C	0.9800
N1—C3	1.278 (3)	C11—H11A	0.9800
C1—C2	1.509 (3)	C11—H11B	0.9800
C1—C10	1.502 (3)	C11—H11C	0.9800
C3—C4	1.438 (3)	C12—H12A	0.9800
C4—C9	1.401 (3)	C12—H12B	0.9800
C4—C5	1.406 (3)	C12—H12C	0.9800
C5—C6	1.366 (3)		
			
O1—Mo1—O2	152.13 (6)	C4—C9—C8	119.30 (19)
O1—Mo1—O4	97.29 (7)	O2—C9—C4	123.2 (2)
O1—Mo1—O5	96.90 (7)	O2—C9—C8	117.52 (18)
O1—Mo1—O6	79.45 (6)	O1—C1—H1	109.00
O1—Mo1—N1	75.32 (6)	C2—C1—H1	109.00
O2—Mo1—O4	97.93 (7)	C10—C1—H1	109.00
O2—Mo1—O5	101.30 (7)	N1—C2—H2A	110.00
O2—Mo1—O6	81.43 (6)	N1—C2—H2B	110.00
O2—Mo1—N1	80.20 (6)	C1—C2—H2A	110.00
O4—Mo1—O5	105.61 (7)	C1—C2—H2B	110.00
O4—Mo1—O6	169.64 (6)	H2A—C2—H2B	109.00
O4—Mo1—N1	95.01 (7)	N1—C3—H3	118.00
O5—Mo1—O6	84.60 (6)	C4—C3—H3	118.00
O5—Mo1—N1	158.82 (7)	C4—C5—H5	120.00
O6—Mo1—N1	74.68 (6)	C6—C5—H5	120.00
Mo1—O1—C1	118.70 (13)	C5—C6—H6	120.00
Mo1—O2—C9	136.56 (13)	C7—C6—H6	120.00
C8—O3—C11	117.57 (18)	C6—C7—H7	119.00
Mo1—O6—C12	128.21 (14)	C8—C7—H7	119.00
C12—O6—H6O	107.7 (18)	C1—C10—H10A	109.00
Mo1—O6—H6O	116.1 (19)	C1—C10—H10B	109.00
Mo1—N1—C3	128.57 (15)	C1—C10—H10C	109.00
Mo1—N1—C2	111.65 (13)	H10A—C10—H10B	109.00
C2—N1—C3	119.75 (19)	H10A—C10—H10C	109.00
O1—C1—C10	110.63 (19)	H10B—C10—H10C	110.00
O1—C1—C2	106.47 (18)	O3—C11—H11A	109.00
C2—C1—C10	112.78 (19)	O3—C11—H11B	109.00
N1—C2—C1	106.57 (17)	O3—C11—H11C	110.00
N1—C3—C4	124.3 (2)	H11A—C11—H11B	109.00
C3—C4—C9	122.31 (19)	H11A—C11—H11C	109.00
C3—C4—C5	117.72 (19)	H11B—C11—H11C	109.00
C5—C4—C9	119.9 (2)	O6—C12—H12A	109.00
C4—C5—C6	120.3 (2)	O6—C12—H12B	109.00
C5—C6—C7	120.1 (2)	O6—C12—H12C	110.00
C6—C7—C8	121.1 (2)	H12A—C12—H12B	109.00
O3—C8—C9	115.41 (19)	H12A—C12—H12C	109.00
C7—C8—C9	119.3 (2)	H12B—C12—H12C	109.00
O3—C8—C7	125.3 (2)		
			
O2—Mo1—O1—C1	−55.3 (2)	Mo1—O2—C9—C4	−21.0 (3)
O4—Mo1—O1—C1	67.30 (14)	Mo1—O2—C9—C8	160.43 (15)
O5—Mo1—O1—C1	174.09 (14)	C11—O3—C8—C7	0.7 (3)
O6—Mo1—O1—C1	−102.75 (14)	C11—O3—C8—C9	−179.1 (2)
N1—Mo1—O1—C1	−25.99 (14)	Mo1—N1—C2—C1	28.0 (2)
O1—Mo1—O2—C9	53.8 (3)	C3—N1—C2—C1	−150.1 (2)
O4—Mo1—O2—C9	−68.64 (19)	Mo1—N1—C3—C4	9.8 (3)
O5—Mo1—O2—C9	−176.39 (19)	C2—N1—C3—C4	−172.5 (2)
O6—Mo1—O2—C9	100.92 (19)	O1—C1—C2—N1	−46.5 (2)
N1—Mo1—O2—C9	25.11 (19)	C10—C1—C2—N1	−168.03 (19)
O1—Mo1—O6—C12	151.18 (18)	N1—C3—C4—C5	−179.5 (2)
O2—Mo1—O6—C12	−8.45 (18)	N1—C3—C4—C9	4.4 (3)
O5—Mo1—O6—C12	−110.76 (18)	C3—C4—C5—C6	−177.1 (2)
N1—Mo1—O6—C12	73.67 (18)	C9—C4—C5—C6	−0.9 (3)
O1—Mo1—N1—C2	−3.26 (14)	C3—C4—C9—O2	−1.2 (3)
O1—Mo1—N1—C3	174.7 (2)	C3—C4—C9—C8	177.28 (19)
O2—Mo1—N1—C2	163.30 (16)	C5—C4—C9—O2	−177.24 (19)
O2—Mo1—N1—C3	−18.79 (19)	C5—C4—C9—C8	1.3 (3)
O4—Mo1—N1—C2	−99.50 (15)	C4—C5—C6—C7	0.1 (4)
O4—Mo1—N1—C3	78.4 (2)	C5—C6—C7—C8	0.3 (4)
O5—Mo1—N1—C2	67.3 (3)	C6—C7—C8—O3	−179.7 (2)
O5—Mo1—N1—C3	−114.8 (2)	C6—C7—C8—C9	0.1 (4)
O6—Mo1—N1—C2	79.59 (15)	O3—C8—C9—O2	−2.5 (3)
O6—Mo1—N1—C3	−102.5 (2)	O3—C8—C9—C4	178.94 (19)
Mo1—O1—C1—C2	49.6 (2)	C7—C8—C9—O2	177.7 (2)
Mo1—O1—C1—C10	172.42 (14)	C7—C8—C9—C4	−0.9 (3)

### Hydrogen-bond geometry (Å, °)

**Table:**

*D*—H···*A*	*D*—H	H···*A*	*D*···*A*	*D*—H···*A*
O6—H6O···O1^i^	0.83 (3)	1.81 (3)	2.639 (2)	176 (2)
C3—H3···O4^ii^	0.95	2.41	3.327 (2)	162
C3—H3···O5^ii^	0.95	2.57	2.958 (3)	105
C10—H10A···O4^iii^	0.98	2.52	3.221 (3)	128
C10—H10B···O5^i^	0.98	2.47	3.407 (3)	161
C11—H11C···O3^iv^	0.98	2.52	3.280 (3)	134

Symmetry codes: (i) −*x*, −*y*, −*z*; (ii) *x*+1, *y*, *z*; (iii) *x*, −*y*+1/2, *z*−1/2; (iv) −*x*, −*y*, −*z*+1.
